# Measuring Internet Gaming Disorder and Gaming Disorder: A Qualitative Content Validity Analysis of Validated Scales

**DOI:** 10.1177/10731911211055435

**Published:** 2021-11-02

**Authors:** Veli-Matti Karhulahti, Marcel Martončik, Matúš Adamkovič

**Affiliations:** 1 University of Jyväskylä, Finland; 2 University of Presov, Slovakia; 3 Institute of Social Sciences, CSPS Slovak Academy of Sciences, Slovakia; 4 Charles University, Prague, Czechia

**Keywords:** addiction, content validity, gaming disorder, qualitative assessment, semantic analysis, technology use

## Abstract

Numerous instruments have been developed to measure gaming-related health problems based on “internet gaming disorder” (IGD) in the third section of the *Diagnostic and Statistical Manual of Mental Disorders* (5th ed.) and “gaming disorder” (GD) in the International Classification of Diseases (11th rev.). However, the criteria in the manuals tend to be operationalized in numerous diverse ways, which can make screening outcomes incomparable. A content validity analysis is needed to reassess the relationships between the diagnostic criteria and the items that operationalize them. The IGD and GD criteria were divided into sematic components. A qualitative content validity analysis was carried out for all items employed by the 17 instruments that claim to measure either construct by their criteria in English. In all but one instrument, the operationalizations did not include all criterion components. There were two main reasons found for this: the components had simply been left out or had been alternatively modified into other components. Criteria that were vaguely described in the manuals were sources of lower content validity items. The study implies that many of the problems in IGD and GD measurement derive from criteria operationalization and original manual descriptions. The conclusion provides practical recommendations that researchers can apply to improve the content validity of their measurement.

After the American Psychiatric Association’s (APA, 2013) call for further research on addictive gaming behaviors in the *Diagnostic and Statistical Manual of Mental Disorders* (5th ed.; *DSM-5*), new self-report survey instruments started to be developed to measure the phenomenon according to the nine criteria listed in the manual. As the World Health Organization (WHO) decided to include gaming disorder (GD) in the 11th revision of the International Classification of Diseases (*ICD-11*) (WHO, 2018) five years later, the same re-occurred with three new criteria listed in the *ICD-11*. These instruments now belong to the 32 validated screening tools for gaming-related problems (King, Chamberlain, et al., 2020), which previously evolved outside diagnostic manuals. Given this large number of completely different instruments and the researchers’ tendency to keep creating and using their own instruments (Toothbrush problem; Mischel, 2008), content heterogeneity has become a serious problem in the field (Costa & Kuss, 2019).

In order to explain and find solutions to this problem, a qualitative content validity analysis was carried out with the items employed by all 17 instruments that currently claim to measure either internet gaming disorder (IGD; *DSM-5* by the APA) or GD (*ICD-11* by the WHO) according to their official criteria. As the rest of the instruments that were found did not claim to measure the APA’s or the WHO’s criteria—derived from numerous other (often undefined or vague) sources—it would have been impossible to reliably assess their content validity against those mixed sources within the limits of this article. Thus, they are not included in this study.

## Importance of Operationalization and Content Validity

The operability of screening instruments depends on one’s philosophy of psychological constructs. If the starting point is that mental disorders are defined by specific sets of indicators or symptoms (see Fried, 2017b), it is important that screening instruments operationalize the right indicators or symptoms. On the contrary, if one’s position is practical and the ontology of mental disorders is not dependent on distinct indicators or symptoms, it matters less what the items in the screening instrument are (i.e., whether they concord with a diagnostic manual) if they reliably identify people whom expert clinicians confirm to suffer from the measured disorder (see Kendler et al., 2011). Although this pragmatic position is popular, for instance, in the measurement of depression, the fact remains that different instruments—with their different items—are *differently* multidimensional (Van Loo et al., 2012), and thus their cut-offs are likely to identify *different* people (Zhao et al., 2017). Ultimately, “rating scales may only be interchangeable indicators of depression severity inasmuch as their item content overlaps” (Fried, 2017a, p. 192).

Unlike chemical elements and other “natural kinds” that natural scientists unanimously agree on, mental disorders are not (currently) definable by clear organic structures (Adam, 2013, but see also Insel et al., 2010). Rather, the mental disorder definitions that are commonly applied by scientists derive from empirically informed views of various experts. These views are not limited to the expert groups which represent the *DSM* and the ICD, however. One of the most popular screening instruments for depression, the Beck Depression Inventory-II (BDI-II) (Beck et al., 1996), is based on Beck’s own theory of depression (Beck et al., 1979), which is different from those in the *DSM-5* and the *ICD-11* (or their previous versions, respectively). Accordingly, scholars using different screening instruments and theoretical foundations may apply similar terminology (e.g., “depression” or “gaming addiction”), but whether their findings are interchangeable still depends on the consistency between the compared constructs, measures, and their empirical outcomes.

Due to the lack of clinical validation studies regarding screening instruments for gaming-related problems (the *ICD-11* diagnosis comes into clinical use in 2022), the diversity of such instruments evokes uncertainty among researchers and clinicians (King & Delfabbro, 2018). For instance, numerous large systematic meta-reviews and meta-analyses (Costa & Kuss, 2019; King, Chamberlain, et al., 2020; Paulus et al., 2018; Richard et al., 2020; Stevens et al., 2021) mix instruments that measure different ideas of gaming-related problems, from pathological gambling-based “internet addiction” to general “addiction components,” which differ in content from both the *DSM-5* and the *ICD-11* criteria. Indeed, reviews of the findings have been found to be systematically unreliable, for instance, due to analysing “studies that used Internet addiction questions to measure gaming disorder” (Colder Carras et al., 2020, p. 14).

Even when construct criteria reach local consensus among scholars who collectively follow the *DSM-5* or *ICD-11*, the instruments still operationalize those criteria in entirely different ways. An example of this is the sixth criterion of “internet gaming disorder” listed in the *DSM-5*, *continued excessive use* (“Continued excessive use of internet games despite knowledge of psychosocial problems”). This been operationalized by respective validated survey instruments as (italics added) follows:

1. “Have you continued your gaming activity despite knowing it was *causing problems between you and other people*?” (IGDS9-SF)
2. “Did you continue to play even though it *created problems for you*? (IGD Scale)
3. “I believe my gaming is *negatively impacting* on important areas of my life.” (IGD-20)

Each of these above operationalizations inquire about different problems: either “problems between you and other people”, “problems for you” or just “negatively impacting” (e.g., school grades) without needing to cause problems at all—or psychosocial problems, as the *DSM-5* criterion states. In order to collect comparable evidence across demographics and populations, the first step should be to operationalize items so that their content is consistent and valid in terms of the described criteria. This is not a trivial task for those who claim to measure the *DSM-5* or the *ICD-11* criteria, as both manuals further elaborate on their concepts by descriptions that are not listed as criteria (e.g., “gaming behavior and other features are normally evident over a period of at least 12 months in order for a diagnosis to be assigned” in the *ICD-11*). Therefore, high content validity (in terms of equivalence) between the items and the listed criteria may not automatically mean high content validity between the instrument and the whole disorder—but it is the critical basis of instrument-specific content validity. Items that do not directly measure the criteria that they claim to measure may be psychometrically valid and useful for screening. However, in such a case, low content validity implies that they do not actually measure what they claim to measure (e.g., GD as defined by the *ICD-11*).

Gaming is a uniquely challenging activity to measure given that millions of people around the world passionately play digital games. It is not straightforward to distinguish between highly passionate play and that which should be diagnosed as a disorder. Indeed, many of the “symptoms” suggested in (and outside of) diagnostic manuals tend to apply to both passionate and problematic habits (e.g., Deleuze et al., 2018; Nielsen & Karhulahti, 2017). The current study does not assess whether the *DSM-5* and the *ICD-11* have chosen the “right” criteria and symptoms for gaming-related problems. Nor does it get involved in wider debates regarding the inclusion of gaming-related problems into diagnostic manuals (see, for example, Dullur & Starcevic, 2018; Enevold et al., 2018; van Rooij et al., 2018). Rather, it focuses on assessing how the screening instruments measure those criteria that they claim to measure. Regardless of whether the manuals’ criteria are “right” or not, the research that employs and measures those criteria should be assessed against them to better understand the relationships between the screening instruments and the screened constructs. A comprehensive understanding of these relationships is a fundamental requirement for being able to properly interpret the results produced by the instruments.

As an example of the current interpretive difficulties, some authors have suggested the criteria and/or symptoms of gaming-related problems to differ in clinical importance, leading to various distinctions between “core” and “peripheral” criteria (e.g., Deleuze et al., 2017; King, Haagsma, et al., 2013; Snodgrass et al., 2019). The fact that different criteria and symptoms are suggested by different authors may lie, at least partially, in how they are (differently) operationalized across the instruments that scholars have used in their reference studies. For example, when the first symptom in the *DSM-5*, “preoccupation,” is measured only by the first part of the symptom (“individual thinks about previous gaming activity or anticipates playing the next game”), the potential “core” or “peripheral” role of this symptom would logically be different from those who measure the *DSM-5*’s second part of preoccupation (“internet gaming becomes the dominant activity in daily life”). As the following analysis shows, these kinds of radical differences in operationalization are very common in the measurement of both *DSM-5-* and *ICD-11*-based gaming problems. With few exceptions, if any, new screening instruments are directly based on the criteria and symptoms in the manuals. However, as their operationalizations are dissimilar, it is unlikely that their outcomes are strongly related to the same constructs.

In order to illustrate these conceptual differences, a recent study that interviewed 100 individuals who had sought treatment for gaming-related problems with both *DSM-5* (IGD) wording and *ICD-11* (GD) wording found the former identified 61 positive results while the latter only showed 36 (Starcevic et al., 2020). Thus, criteria and wording differences do matter significantly, both between and within concepts. To understand the differences within the IGD and GD instruments, this article focuses on their semantic criteria-item relations.

To the best of our knowledge, there has been no previous content validity studies done on IGD and GD. In a comprehensive systematic review by King, Chamberlain, et al. (2020), the consistency of individual measures was assessed and some instruments found to have greater evidential support. However, their highest ranked instrument, Game Addiction Scale (GAS-7), was created before both the IGD and GD diagnostic criteria, thus making it conceptually different (i.e., not directly measuring *DSM-5* or *ICD-11* dimensions). Similarly, a face validity study by King, Billieux, et al. (2020) “provided support for the items contained in Petry et al. (2014) IGD measure [whose] items were adequately aligned with the *DSM-5* criteria” (p. 10). However, an analytical look at the instrument’s first item (“Do you spend a lot of time thinking about games even when you are not playing, or planning when you can play next?”) already reveals that it is not strongly connected to the corresponding *DSM-5* criterion of gaming being a “dominant activity in daily life” (recall above; see Griffiths et al., 2016). Therefore, an in-depth qualitative content validity analysis is needed to properly reassess the semantic relationships between the official diagnostic criteria and the items that claim to operationalize them.

## Method

### Review of Instruments

For this review, all instruments were selected that explicitly claim to measure IGD (“internet gaming disorder” in the *DSM-5*) or GD (“gaming disorder” in the *ICD-11*). In addition to the nine instruments that were found in the systematic review by King, Chamberlain, et al. (2020), a database search was conducted which yielded eight more instruments, making it 17 in total. One of the instruments (Petry et al., 2014) merely *suggested* operationalizations for IGD, but was later validated (Jeromin et al., 2016) and has been frequently used by scholars under the name “Internet Gaming Disorder Checklist” (e.g., Borges et al., 2019; Deleuze et al., 2017; King & Delfabbro, 2016; Schneider et al., 2017). In a similar way, IGD–Brief Indicators Checklist (IGD-BIC) (Przybylski et al., 2017) was developed specifically based on IGD and has been used in previous studies (e.g., Przybylski, 2016; Weinstein et al., 2017). However, we were not able to find a validation study for it. All the studies are summarized in Table 1.

**Table 1.** Description of Reviewed Measures That Are Based on Either the *DSM-5* or *ICD-11* Approach.

Scale name	Measured construct	Number of items	Response format	Covers the period of last 12 months	I/GD criteria cut off
Internet Gaming Disorder Checklist, IGDC (Petry et al., 2014)	IGD	9	NA	Not stated.	≥5 criteria
Internet Gaming Disorder 20 Test, IGD-20 (Pontes et al., 2014)	IGD	20	5-point Likert scale (*strongly disagree; disagree; neither agree or disagree; agree; strongly agree*)	Yes	≥5 criteria
Internet Gaming Disorder Scale, IGDS (Lemmens et al., 2015)	IGD	9/27	(1) Yes/no; (2) 6-point ordinal scale (never; 1*–4 times in the last year; 5–11 times in the last year; about 1–3 times a month; once or more a week; every day or almost every day*)	Yes	≥5 criteria (*DSM*-5 approach) or >6 criteria (supported with the Latent Class Analysis)
Internet Gaming Disorder Scale–Short-Form, IGDS9-SF (Pontes & Griffiths, 2015)	IGD	9	5-point ordinal scale (*never; rarely; sometimes; often; very often*)	Yes	Cut-off score of 36
Video Game Dependency Scale, CSAS (Rehbein et al., 2015)	IGD	18	4-point Likert scale (*strongly disagree; somewhat disagree; somewhat agree; strongly agree*)	Yes	≥5 criteria
Personal Internet Gaming Disorder Evaluation-9, PIE-9 (Pearcy et al., 2016)	IGD	9	5-point ordinal scale (*never* to *very often*)	Yes	≥5 criteria
Ten-Item Internet Gaming Disorder Test, IGDT-10 (Király et al., 2017)	IGD	10	3-point ordinal scale (*never; sometimes; often*)	Yes	≥5 criteria (supported with the Latent Class Analysis)
Internet Gaming Disorder–Brief Indicators Checklist, IGD-BIC (Przybylski et al., 2017)	IGD	9	Yes/no	Yes	≥5 criteria
CVAT 2.0 (van Rooij et al., 2017)	IGD	11 (9 *DSM*-5-compliant items + 2 legacy C-VAT items)	Yes/no	Yes	≥ 5 criteria for the 9 *DSM*-5 items
Chinese Internet Gaming Disorder Scale, C-IGDS (Sigerson et al., 2017)	IGD	9	Yes/no	Yes	NA
Internet Game Use-Elicited Symptom Screen, IGUESS (Jo et al., 2017)	IGD	9	4-point ordinal scale (*not at all; occasionally; frequently; always*)	NA	Cut-off score of 10 (based on ROC curve analysis)
IGD scale (Finserås et al., 2019)	IGD	9	Yes/no	Yes	NA
IGDS-23 (Borges et al., 2019)	IGD	23	Yes/no	Yes	≥5 criteria
Gaming Disorder Test, GDT (Pontes et al., 2021)	GD	4	5-point ordinal scale (*never; rarely; sometimes; often; very often*)	Yes	Score of at least 4 on each of the four items
Gaming Disorder Scale for Adolescents, GADIS-A (Paschke et al., 2020)	GD	10	5-point Likert scale (*strongly disagree; somewhat disagree; partially agree*/*partially disagree; somewhat agree; strongly agree*)	Yes	>12.5
Gaming Disorder and Hazardous Gaming Scale, GDHGS (Balhara et al., 2020)	GD	4	5-point ordinal scale (*never; once or twice; monthly; weekly*; and *daily or almost daily*)	Yes	All criteria
The Three-Item Gaming disorder Test–Online-Centered, TIGTOC (Jo et al., 2020)	GD	3	4-point ordinal scale (*not at all; occasionally; frequently; always*)	Yes	Score of 4 or higher

*Note.* Items used in IGD-BIC (Przybylski et al., 2017) are available elsewhere at: https://osf.io/a9ya2/. *DSM*-5 = *Diagnostic and Statistical Manual Of Mental Disorders* (5th ed.); *ICD-11* = International Classification of Diseases, 11th Revision; IGD = internet gaming disorder; NA = information not provided in the manuscript; CVAT = The Clinical Video game Addiction Test; GD = gaming disorder.

With the noted exceptions, all the analysed instruments have been validated (in many ways) in peer-reviewed scientific journals and published in English. Six of the 17 instruments have been published in English despite being validated with non-English-speaking samples via a corresponding native language version. This applies to the Dutch CVAT (The Clinical Video game Addiction Test 2.0) (van Rooij et al., 2017), Chinese C-IGDS (Chinese Internet Gaming Disorder Scale) (Sigerson et al., 2017), Korean IGUESS (Internet Game Use-Elicited Symptom Screen) (Jo et al., 2017), Norwegian IGD scale (Finserås et al., 2019), Spanish IGDS-23 (Internet Gaming Disorder Scale-23) (Borges et al., 2019), and German GADIS-A (Gaming Disorder Scale for Adolescents) (Paschke et al., 2020). As the studies have already been applied to English-speaking samples—albeit not always validated in English—these were included in our analysis. Moreover, four other instruments lacked explicit statements about their original language. As they were used in samples of adolescents and young adults (12+ years old) from countries that did not have English as their official language, it is possible that these four instruments also represent English instruments that have been validated in non-English languages. These four instruments are the IGDS (Lemmens et al., 2015) that was validated with a sample of Dutch adults and adolescents, the Video Game Dependency Scale (CSAS) (Rehbein et al., 2015) that was validated with a sample of German adolescents, the 10-Item Internet Gaming Disorder Test (IGDT-10) (Király et al., 2017) that was validated with a sample of Hungarian gamers, and the Three-Item Gaming Disorder Test–Online-Centered (TIGTOC) (Jo et al., 2020) that was validated with a sample of Korean adolescents.

When choosing instruments for measurement, Groth-Marnat and Wright (2016, p. 11) encourage all clinicians to ask, “Do the test items correspond to the theoretical description of the construct?” Accordingly, the current study analysed each criterion in relation to how they have been operationalized verbally in each of the instruments. To be clear, space did not allow the full content validity to be assessed—to what extent all the instruments’ items measure the constructs known as IGD and GD (assessment of content domain) by the whole range of behavioral manifestations in the manuals, typically done separately by several external subject matter experts. Indeed, the current goal is not to discuss what constitutes IGD or GD let alone evaluate the overall quality of screening tools, but rather to assess how well the currently applied items operationalize the criteria that the APA and the WHO propose as essential in their respective manuals.

### Review Process

The study did not require local ethics committee reviews. The scale items were assessed by each author separately. None of the authors have been involved in the development of any of the IGD or GD constructs (in the APA or the WHO) nor in the validation of the related screening instruments, thus allowing us to carry out the assessment without conflicts of interest. A degree of concordance between the description of specific criterion and respective item operationalization was assessed using a judgmental method typically used in evaluations of test content (Aiken, 1980). Each *DSM-5* criterion and *ICD-11* criterion were semantically divided into components and the content validity rating was assigned to all items based on how many of the identified criterion-specific components they considered. Thus, each item was evaluated and its evidence of content-related validity rated on a 3-point scale ranging from low validity to moderate and high validity. Eventhough the overall inter-rater agreement was good (intraclass correlation coefficient [ICC] = .80), several items had to be iteratively discussed until a consensus was agreed. The code, data, and ratings are available at https://osf.io/qax5r/.

## Results

Detailed results of the qualitative analysis are available in Supplement 1, and a summary is presented in Table 2. The three main findings follow below.

**Table 2.** Concordance Between the Description of Criteria in Diagnostic Manuals and Their Operationalization in Scales.

	*DSM-5* criteria									ICD 11 criteria		
Scale	1	2	3	4	5	6	7	8	9	1	2	3
IGDC	◑	○	○	◑	◑	◑	◑	◑	●			
IGD-20	◑	◑	●	◑	●	○	◑	○	○			
IGDS	◑	◑	◑	◑	◑	○	●	◑	◑			
IGDS9-SF	●	○	◑	●	◑	◑	●	●	●			
CSAS	◑	○	◑	◑	◑	◑	◑	◑	◑			
PIE-9	◑	◑	●	●	◑	◑	●	●	●			
IGDT-10	◑	◑	◑	◑	○	◑	●	◑	◑			
IGD-BIC	◑	◑	◑	○	○	○	●	◑	○			
CVAT 2.0	◑	◑	●	◑	◑	○	◑	○	○			
C-IGDS	◑	●	◑	●	●	●	●	●	●			
IGUESS	◑	○	◑	●	◑	●	●	◑	◑			
IGD Scale	◑	◑	◑	◑	●	○	◑	◑	●			
IGDS-23	◑	○	○	◑	◑	◑	●	○	●			
GDT										●	●	●
GADIS-A										◑	◑	◑
GDHGS										●	◑	◑
TIGTOC										◑	◑	◑

*Notes.* DSM-5 = Diagnostic and Statistical Manual of Mental Disorders (5th ed.); IGDC = Internet Gaming Disorder Checklist; ◑ = moderate validity; ○ = low validity; ● = high validity; IGD-20 = Internet Gaming Disorder-20 Test; IGDS = Internet Gaming Disorder Scale; IGDS9-SF = Internet Gaming Disorder Scale–Short-Form; CSAS = Video Game Dependency Scale; PIE-9 = Personal Internet Gaming Disorder Evaluation-9; IGDT-10 = Ten-Item Internet Gaming Disorder Test; IGD-BIC = Internet Gaming Disorder–Brief Indicators Checklist; CVAT 2.0 = Clinical Video game Addiction Test 2.0; C-IGDS = Chinese Internet Gaming Disorder Scale; IGUESS = Internet Game Use-Elicited Symptom Screen; IGD = internet gaming disorder; IGDS-23 = Internet Gaming Disorder Scale-23; GDT = Gaming Disorder Test; GADIS-A = Gaming Disorder Scale for Adolescents; GDHGS = Gaming Disorder and Hazardous Gaming Scale; TIGTOC = Three-Item Gaming disorder Test–Online-Centered.

### Criterion Components

Each criterion was semantically divided into central components. A component is a behavioral manifestation, and as such represents content of interest. It was found that most of the questionnaires omit at least one important component. For example, the first *DSM-5* criterion, *preoccupation*, included three components: (a) thinking about previous gaming (*DSM-5*: “the individual thinks about previous gaming activity”), (b) anticipating future gaming (*DSM-5*: “or anticipates playing the next game”), and (c) gaming becomes a dominant activity (*DSM-5*: “internet gaming becomes the dominant activity in daily life”). All three criteria are captured by only one instrument (Internet Gaming Disorder-20 Test [IGD-20]). Most of the reviewed instruments clearly focus on (a) and (b) but ignore (c) entirely.

### Modifying Components

Coining new wording for a criterion component easily transforms its meaning and the possible range of interpretations. This was found to be the most frequent problem in criterion operationalization. For instance, the second criterion listed in the *DSM-5*, *withdrawal*, is worded to apply to situations when internet gaming is “taken away,” namely, forced removal of the gaming activity. While some instruments (e.g., CSAS, IGDS) modify this to gaming being unavailable, others inquire about personal attempts to reduce gaming time or stopping gaming entirely (e.g., Internet Gaming Disorder Checklist [IGDC], Internet Gaming Disorder Scale–Short-Form [IGDS9-SF]) or less play (e.g., IGDT-10). Another example is the ninth *DSM-5* criterion, *jeopardizing or losing a significant relationship, job, or educational or career opportunity*, which is sometimes operationalized merely as “problem” in the previously mentioned domains (e.g., CVAT 2.0). Alternatively, the IGDT-10 has modified jeopardizing and loss of work into a question of gaming affecting work performance while the IGDS-23 asks about risking an opportunity at school or work (including e.g., an opportunity to have dinner with a colleague).

### Replacing Examples

Some of the criteria listed in the manuals include examples. For instance, the second *DSM-5* criterion, *withdrawal*, names typical symptoms: irritability, anxiety, and sadness. These typical symptoms are often replaced by feelings of stress, annoyance, anger, frustration, restlessness, worry or sadness (e.g., in IGDS-23, Personal Internet Gaming Disorder Evaluation-9 [PIE-9], or CVAT 2.0). Although “typicality” as an official term in the manual opens the possibility for scholars to inquire about “less typical” possible symptoms as well, the great diversity of symptoms worded by different instruments contributes to their incoherence. If the instruments did not replace the examples provided by the *DSM-5* and *ICD-11* with their own examples, the results they produce would be more comparable and coherent with the measured content.

## Discussion

In the literature on gaming-related problems, the most systematic finding of the systematic reviews (e.g., Colder Carras et al., 2020; Costa & Kuss, 2019; King, Chamberlain, et al., 2020) is perhaps that the field operates with a great range of heterogeneous and inconsistent survey instruments. As this “diagnostic confusion” (Pontes & Griffiths, 2014) already characterized the field at the time of the *DSM-5*’s release (King, Haagsma, et al., 2013), the descriptions provided by the *DSM-5* (IGD) and the *ICD-11* (GD) were important as an opportunity to make the field more consistent and coherent, at least in terms of operationalized criteria. Alas, the current content analysis of all available instruments that claim to measure IGD or GD suggests that few tools correspond with the criteria of content that the APA and the WHO have outlined. One key reason for this is pragmatics—conveying all content provided by the criteria listed in the diagnostic manuals is difficult and heavy to operationalize. However, this is hardly the only reason, considering the extreme degree of heterogeneity. The fact that instrument creators make diverse component inclusion decisions, modify criteria components and replace given examples echoes more general challenges related to the development of screening tools. Part of these challenges derive from how the *DSM-5* and the *ICD-11* have decided to express their IGD and GD criteria (not to be confused with the general criticism of these criteria).

In terms of content validity, the *DSM-5* Criteria 2 (*withdrawal*) and 6 (*continued excessive use*) were found to be the most difficult to operationalize while Criterion 7 (*deception*) was found to be the easiest to operationalize. It is possible to explain this after taking a closer look at their wordings. Criteria 2 and 6 contain abstract constructs—withdrawal symptoms, psychosocial problems—that remain undefined and vague. The lacking clarity has resulted in substitutions of various kinds (e.g., CVAT 2.0, IGDS, IGDS-23, PIE-9, IGD Scale). For instance, the concept “use of games” in Criterion 6 is often narrowly operationalized as “playing games” and thus ignores watching games, socializing in games, and learning new gaming strategies, among other uses of especially competitive esports games (Karhulahti, 2020). Whether or not this has been the intention of the APA group, such wording points at behavioral manifestations that are not operationalized in the instruments as such. On the contrary, the most accurately operationalized criterion does not contain abstract constructs: *deception* related to the amount of play and enumeration of all persons affected by the deception is unambiguous. If other criteria consisted of such unequivocal descriptions as well, the overall content validity of the field would likely be higher. On the other hand, gaming-related problems can be diverse and may not be easily turned into clearly measurable criteria or symptoms. Future clinical research should pursue a better understanding of what these problems are and how they manifest. Following this, reliable screening instruments can be developed.

Different operationalizations, or item wordings, then raise the question as to whether the instruments measure the same construct or if several constructs are being measured. The latter is supported by the fact that different screening tools currently produce different prevalence rates (Darvesh et al., 2020; Mihara & Higuchi, 2017). It would be useful to compare all assessed instruments by their prevalence rates—although this is not currently possible since the study designs and samples differ greatly. It is not known if present prevalence rate differences are caused by study designs, operationalizations or other reasons.

Indeed, it is also possible that different types of playing (in different life contexts) constitute numerous overlapping constructs. For instance, one of the few in-depth clinical case studies has suggested two different pathways of IGD (Benarous et al., 2019), whereas survey-based clustering (Billieux et al., 2015) and latent class analyses (Carras & Kardefelt-Winther, 2018) have identified five subtypes (only some of which were considered clinically significant). Furthermore, one literature-based typology (Lee et al., 2017) has suggested four types of clinically significant play. Although none of these typologies have been replicated or triangulated via other methods as far as we are aware, future instrument development should follow a thorough examination of the nature of the construct(s), whether single or plural.

Finally, a study like this should ask to what degree IGD and GD themselves deserve to be treated as separate constructs. It is probable that our descriptions and understandings of (internet) GD, as it has evolved over the years, keep evolving still along with new editions of the *DSM* and the ICD. Even an instrument with “perfect” content validity, in terms of the present notion of (internet) GD, would eventually become outdated due to the persistently evolving disorder criteria. In such scenarios, it is only natural that old instruments become incomparable to new instruments. Before these imminent changes occur, it is recommended for the instruments to pursue content validity if their goal is to measure the current criteria in the available diagnostic manuals.

### Limitations

This qualitative study was limited by the diversity and size of the team. Although the items were assessed systematically based on explicit criteria components and their correspondences documented in detail, it is possible that a more diverse and larger research team would have ended up with different results and conclusions. That said, as an upshot of this study, an open commentary space has been initiated to further crowdsource and pursue consensus over the content validity of the currently available instruments as well as those that will be published later (https://osf.io/t9u5p/). We look forward to improving the current findings as a wider scientific community in open scientific dialogue.

Secondly, the study was limited to English. Several IGD and GD instruments have been developed in and translated to/from other languages. Their content validity should be assessed separately. Thirdly, it is necessary to acknowledge the challenges related to content validity analysis in relation to GD and IGD. As for the latter, the *DSM-5* lists nine explicit criteria, but expands on those criteria elsewhere in the manual so that any researcher with the goal of creating an instrument based on these descriptions is forced to compromise. Although the available instruments have been analysed in light of the criteria that they represent, we are also sympathetic to the instrument creators’ limited possibilities of accurately and efficiently reproducing the content that the *DSM-5* offers. To a degree, the same concerns GD in the *ICD-11*, which lists three explicit criteria, but soon also adds that disordered gaming behavior “may be continuous or episodic and recurrent.” Further clarity in the diagnostic manuals would significantly help clinicians and scholars pursue consensus in screening.

Lastly, as previously highlighted, the study was limited to the instruments that claim to measure IGD (in the *DSM-5*) and GD (in the *ICD-11*), and instruments that measure gaming-related problems according to other models or theories were not included. Such instruments may have high content validity in relation to their own foundations.

### Recommendations

1. *If you intentionally measure only part of the criteria listed in a diagnostic manual, state this explicitly*. Instrument creation always involves compromises. If such compromises relate to excluding some of the criteria or parts thereof, addressing and motivating these decisions openly help the community in assessing the relationship between the operationalization and the measured construct. If several criteria or criteria components are not included as a deliberate trade-off, it is also possible to describe the instrument as not explicitly measuring a construct by a diagnostic manual.
2. *If you measure the criteria in a diagnostic manual, use synonyms and jargon cautiously*. This especially applies when translating/adapting items to other languages. Many of the instruments that this study reviewed were validated in non-English languages—even though the instruments were published in English. Instruments and their items should always be validated in the language that they are being used. That said, English instruments are often applied to international samples (with participants who are not natives in English). Hence the use of phrases such as “fretting about a game” in IGDS-20 may lead to unreliable responses (see the sixth recommendation).
3. *Use concrete examples and clarifications*. The use of overly simplistic operationalizations like “I have been preoccupied with internet games” may be understood in many ways. When introducing complex and rare terms to participants, it is advised to use examples for better clarification, for example, withdrawal symptoms (such as irritability, anxiety, or sadness) or psychosocial problems (e.g., loneliness or social anxiety). Examples or clarifications are especially important for populations such as children and adolescents who may not know what “psychosocial problems” refer to or what exactly is “preoccupation.” If you measure a criterion in a diagnostic manual, prefer the provided existing examples.
4. *Avoid conditional items*. Conditional items present two distinct statements that require the respondent to typically think in the following way: “I would like to behave like Y, but X is preventing me from doing it.” As such, the respondent must choose one response to two questions. For instance, when IGD-20 states “I would like to cut down my gaming time but it’s difficult to do,” the respondent may be confused about whether they should state their agreement with “I would like to cut down my gaming time” or “it’s difficult to do.” Similarly, IGDS9-SF asks, “Do you systematically fail when trying to control or cease your gaming activity?” and IGDS asks, “did you want to play less, but couldn’t?” Conditional items can be especially troublesome for children, people with cognitive or reading disabilities, and expert researchers. The issue has also been raised by Borges et al. (2019, p. 716) who “preferred here to break down the nine symptoms into short dichotomous questions that use a direct formulation and avoid subclauses.”
5. *Avoid using suggestive or leading questions*. Asking “How often have you. . ..” instead of “I have. . .” can inflate the prevalence rate, as the former implicitly assumes “it” happens. Suggestive items may indicate that responding in frequency is socially more desirable than a negative answer, for example, “How many books did you read last month?” may lead a person to think that they should have read some. Neutral wordings such as “I have. . .” with an ordinal rating scale should generally be used.
6. *Adapt instruments cautiously*. The validity of an original measure does not ensure validity in a translated/adapted version. This also works the other way around: evidence of validity of the adapted version does not guarantee validity of the source. As such, evidence of validity should always be interpreted solely for the language that was used in the validation study. If a researcher aims to provide evidence that both the target and the source (usually English) versions of the instrument operate similarly, in addition to the essential language equivalence, evidence of both construct and measurement equivalence needs to be provided (see Byrne, 2016).

As the reviewed instruments already exist and consensus is difficult to reach (see Griffiths et al., 2016, recall the toothbrush problem), multiple operationalizations should be verified together to find out whether diverse prevalence rates or relationships with variables occur. It is not necessary to abandon all existing instruments for the sake of developing new ones, although new instruments (e.g., Pontes et al., 2021) may allow diagnostic comparability between classification systems (*DSM-5* vs. *ICD-11*). It would be highly beneficial to evaluate the clinical usefulness of both instruments (wordings) aligned with the diagnostic manuals and also those with alternative wordings or questions mapping additional symptoms not covered by the diagnostic manuals. Such a strategy (sensitivity analysis) is already frequently recommended in other fields of science (see, for example, Adamkovič et al., 2020; Fanchamps et al., 2018; Stallinga et al., 2014).

One of the current key challenges in the study of gaming-related problems is the lack of qualitative clinical evidence, as the number of people being treated has been small (compared to anxiety or depression for example). Along with the expectedly increasing number of treatment-seekers in 2022 when GD in the *ICD-11* officially comes into clinical use, it is important to clinically validate the screening instruments. This will allow identifying instruments that are useful in practice by

(a) preventing both Type I and Type II errors that in everyday society can, respectively, cause moral panic and deny help to those who need it, and
(b) facilitating scientific progress by directing researchers to screening instruments that produce reliable results.

As for the latter, this can eventually increase the chance of identifying early warning signals, help tailor more effective interventions through a better understanding of the structure of GD, and save human resources in general.

## Conclusion

Although (internet) GD has only been implemented in the latest versions of the *DSM* (third section) and the ICD diagnostic manuals, there are an abundance of screening tools that assess the phenomenon based on their criteria. However, many of these instruments operationalize the criteria freely, lowering their validity in relation to the referenced content. This produces heterogeneity that leads to varying sensitivities, specificities, and subsequently to incomparable prevalence rates as well as diverse statistical estimates when conducting research. Based on the present validity findings, the study offers practical recommendations for researchers studying (internet) GD to consider (see also general textbook advice, for example, Krosnick & Presser, 2010) when developing/modifying instruments. As such, the recommendations hopefully produce a less fuzzy framework in which to pursue related scientific goals, be they a better understanding of the complexity of the phenomenon or means of its treatment and prevention. A crowdsourcing collaboration of experts on both gaming and psychological measurement—with more in-depth qualitative evidence on the phenomenological nature and identifiable number of symptoms—can eventually produce solutions that bring clarity and consistency into the rather muddy field of measuring gaming-related problems. One such collaboration opportunity is opened as a conclusion of this article and readers are welcome to comment: https://osf.io/t9u5p/.

## Supplemental Material

sj-docx-1-asm-10.1177_10731911211055435 – Supplemental material for Measuring Internet Gaming Disorder and Gaming Disorder: A Qualitative Content Validity Analysis of Validated Scales Click here for additional data file. Supplemental material, sj-docx-1-asm-10.1177_10731911211055435 for Measuring Internet Gaming Disorder and Gaming Disorder: A Qualitative Content Validity Analysis of Validated Scales by Veli-Matti Karhulahti, Marcel Martončik and Matúš Adamkovič in Assessment
