# Supplementary material for: Measuring Internet Gaming Disorder and Gaming Disorder: A Qualitative Content Validity Analysis of Validated Scales
Source: Assessment. 2021 Nov 2;30(2):402–13. doi: 10.1177/10731911211055435 (PMC9900185; doi:10.1177/10731911211055435)
Supplement: sj-docx-1-asm-10.1177_10731911211055435 – Supplemental material for Measuring Internet Gaming Disorder and Gaming Disorder: A Qualitative Content Validity Analysis of Validated Scales [file sj-docx-1-asm-10.1177_10731911211055435.docx]

**Results of the review**

Results are provided separately for all the diagnostic criteria stated in the DSM-5 and the ICD-11 manuals, respectively.

**1. Preoccupation with internet games. The individual thinks about previous gaming activity or anticipates playing the next game; internet gaming becomes the dominant activity in daily life** (IGD in DSM-5)

This criterion can be divided into three: (1) thinking about previous gaming (“the individual thinks about previous gaming activity”), (2) anticipating future gaming (“anticipates playing the next game”), and (3) gaming becomes a dominant activity (“Internet gaming becomes the dominant activity in daily life”). While the first two represent specific cognitive processes, the third is a more general state of affairs. Of note, the emergence of (3) would unlikely be connected *only* to (1) and (2); furthermore, (3) could also include non-gaming activities such as watching other people play games (e.g., live-streams), consuming other gaming-related media (e.g., videos), talking about games (online and offline), attending gaming events (festivals and tournaments), and other such activities.

So how are (1), (2), and (3) operationalized in the 13 instruments that aim at capturing this dimension? Our analysis shows that one instrument operationalizes these criteria somewhat accurately, whereas 12 instruments fall short. For instance, IGDC uses an item “I have been preoccupied with Internet games” and the item in IGD-BIC is “spent too much time thinking about games”, which imply (1) or (2) but do not address the “dominant activity” in a way that (3) states. In fact, most of the reviewed instruments clearly focus on (1) and (2) but ignore (3) entirely. The item crafted for IGUESS further exemplifies: “I kept thinking about the Internet (online) games I used to play or was engrossed in playing them.” As operationalizations like this do not try to capture part (3), they also struggle with both (1) and (2) by not making a clear distinction between the past and the future.

Three instruments can be said to deal with all (1), (2), and (3): IGD-20 Test, IGDS9-SF, and IGDS. The latter includes three unique items: “have there been periods when you were constantly thinking about a game while at school or work?”, “have there been periods when all you could think of was the moment that you could play a game?”, and “have there been periods when you were constantly fretting about a game?” Whereas the first two items match reasonably well with (1) and (2), the third item and its phrasing “fretting about a game” hardly corresponds with the “dominant activity” criterion. A more accurate wording in this regard is the IGDS9-SF, which addresses all (1), (2), and (3) explicitly: “Do you feel preoccupied with your gaming behaviour? (Some examples: Do you think about previous gaming activity or anticipate the next gaming session? Do you think gaming has become the dominant activity in your daily life?)” However, only a single item is employed for asking about all three respective symptoms, meaning that respondents who anticipate a future gaming session are dumped into the same group with those to whom gaming is a dominant life activity, hence making the results representative mainly of the lowest peripheral dominators. Despite mentioning all parts, the item does not operationalize them in practice.

The only instrument that comes very close to meeting all these contents is IGD-20 and its two items: “I usually think about my next gaming session when I am not playing” and “I think gaming has become the most time consuming activity in my life”. These items respectively correspond with (2) and (3), whereas (1) is not addressed. However, if one understands (1) as a peripheral symptom that merely contributes to (3), IGD-20 can be considered highly valid.

**2. Withdrawal symptoms when internet gaming is taken away. These symptoms are typically described as irritability, anxiety, or sadness, but there are no physical signs of pharmacological withdrawal** (IGD in DSM-5)

Since the DSM-5 only lists “typical” symptoms for this criterion, literally any non-physical response to withdrawal applies here. This makes operationalizing this criterion difficult, and one of the possible solutions for doing that would be to employ the listed “typical” symptoms as indicators and leave the rest for interpretation, as the authors of the DSM-5 have decided. Attention must also be put on the words “taken away”, which directly point at forced removal of the gaming activity. Since the goal here is not to evaluate these criteria but the content validity of the instruments that employ them, we now look specifically at how these elements, (1) withdrawal symptoms and (2) “taken away”, have been taken into consideration in the items.

Of the 13 instruments that included this criterion, we found one with high and 6 with moderate content validity. The most common factor that undermined present content validity was the ignorance of (2) or modifying it with alternative scenarios such as personal attempts to reduce gaming time. For instance, the IGDS9-SF operates with the following item: “Do you feel more irritability, anxiety or even sadness when you try to either reduce or stop your gaming activity?” Again, in this item, as in many others, the external forces that “take away” the gaming activity have been switched to the player’s personal attempt to reduce or stop gaming. The CVAT 2.0, in turn, asks: “Did you feel stressed, annoyed, or angry if you were not allowed or could not play games?” In this case, (2) has been replaced with a scenario in which gaming is not available, moreover, the original symptoms (irritability, anxiety, sadness) have been swapped to three new symptoms: feeling stressed, annoyed, or angry. The same applies to IGDS-23 too, as it neglects “taken away” and instead applies three new symptoms: restlessness, worry, and anger.

No more than two instruments include (2): PIE-9 and C-IGDS. The former applies the following item: “I have experienced withdrawal symptoms when Internet gaming is taken away (such as anger, frustration, or sadness).” This is very much in line with the APA’s base wording, however, for some reason the authors have excluded irritability and anxiety and included anger and frustration instead. This is somewhat reasonable since the APA presents their three symptoms as examples (with anger noted outside the list). The question used by C-IGDS is even more faithful: “Do you feel irritable, anxious, or sad when Internet gaming is taken away?” The item does not make it clear that the listed symptoms are examples – e.g. respondents who feel “anger” should answer this question negatively – but otherwise its content validity is high.

**3. Tolerance—the need to spend increasing amounts of time engaged in internet games** (IGD in DSM-5)

First, the decision to address (1) the need to spend time in particular makes clear that players must not necessarily play increasingly but have the need to do so. Second, the decision to speak of (2) *increasing* amounts is comparative, i.e. operationalizations should address a change from the past. Third, the decision to address gaming (3) *engagement* rather than playing games allows, again, the inclusion of watching games, reading about games, and other forms of gaming engagement. Compared to the previously discussed criteria, this is a relatively well operationalized criterion, as many of the instruments include it with a very similar wording.

Two of our reviewed instruments have content validity issues due to (1), as their items have not been built to measure the need for gaming but actual gaming. E.g., CVAT 2.0 directly asks: “Did you spend more and more time on playing videogames?” Likewise, IGUESS employs the following item: “The time I spend on Internet games is getting longer and longer.” Another three reviewed instruments have issues with (2), in turn. IGD-20 has three separate items for this criterion, the last of which is: “I often think that a whole day is not enough to do everything I need to do in-game.” This item does not address increase but rather a stable problematic state of affairs. In a similar way, IGDS employs three unique items, and the last one as follows: “... have you felt unsatisfied because you wanted to play more?” The question does not inquire about increased engagement per se but rather the felt (un)satisfaction related to it. A focus on satisfaction rather than engagement is also present in IGDS-23, “Did you play games longer than you used to, and still not feel excited or satisfied by them?” as well as in IGD-BIC, “increased playtime to keep excitement high.”

Almost all reviewed instruments struggle with (3) in terms of content validity, yet three succeed well. The C-IGDS item asks: “Do you feel the need to engage in Internet games with increasing amounts of time in order to achieve satisfaction?” As far as we know, the DSM-5 does not imply the need in question to be motivated by satisfaction, even though it can be. For instance, the DSM-5 does not exclude the possibility that an increasing need may also be a result of expanding social interaction that cannot be measured as “satisfaction achievements”. Therefore, although all relevant content is included, an extra requirement has been added. Ultimately, we find the content validity of PIE-9 and IGD-20 well in line with the described notion of tolerance: “I find an increasing need to spend increasing amounts of time engaged in Internet games” (former) and “I need to spend increasing amounts of time engaged in playing games” (latter).

**4. Unsuccessful attempts to control the participation in internet games** (IGD in DSM-5) **– Impaired control over gaming (e.g., onset, frequency, intensity, duration, termination, context)** (GD in ICD-11)

Issues of control are the first criterion that is mutual to the DSM-5 and the ICD-11. Visibly, the descriptions between the two are rather different, the former being more limited by addressing (1a) “unsuccessful attempts to control” and not (2a) “impaired control”. Likewise, the DSM-5 speaks of (1b) “participation in internet games” whereas the ICD-11 addresses (2b) “over gaming” more generally with several clarifying factors named. We start by discussing the IGD instruments. The operationalization by IGDC goes as follows: “Do you feel that you should play less, but are unable to cut back on the amount of time you spend playing games?“ IGD-BIC apply a similar item “felt that I should play less but couldn't.” These two do not address the notion of “control” at all but reduce it to “less play” akin to many other instruments: CVAT 2.0 asks “Did you unsuccessfully try to spend less time on games?” and IGDT-10 asks: “Have you ever in the past 12 months unsuccessfully tried to reduce the time spent on gaming?” Specifically, the reduction of time is only one of the many ways to “control”, as also specified by the ICD-11’s “onset, frequency, intensity, duration, termination, context.” For instance, to get proper sleep, one who can control could stop playing in the evening and play more in the morning, thus not reducing overall play time but controlling it.

Three IGD instruments do well in capturing (1a). One of these is C-IGDS, which introduces a double plural: “Have you *repeatedly* made unsuccessful *attempts* to control your participation in Internet games?” (emphasis added). This word choice may deflate positive responses; for instance, two attempts would already be multiple but hardly “repeated”. PIE-9 and IGDS9-SF, in turn, accurately correspond to the APA criterion: “I have had unsuccessful attempts to control the participation in Internet games” and “Do you systematically fail when trying to control or cease your gaming activity?”

We address all four GD instruments here. First, GDHGS captures (2a) and (2b) well: “Do you fail while trying to control or stop your gaming behaviour?” GDT likewise states: “I have had difficulties controlling my gaming activity.” These are valid items and we have no critical comments on them. In TIGTOC, however, (2a) is again reduced to cutting down, similarly as in the DSM-5 instruments above: “I have tried to cut down playing Internet games, but I have not been successful.” GADIS-A likewise reduces control to gaming time and states: “I often play games more frequently and longer than I planned to or agreed upon with my parents.” This item has another problem with an assumption that the respondent has parents with whom they have an agreement about gaming frequency and time. Although the instrument is made for adolescents, such assumptions hurt the item’s content validity by excluding all respondents without parents as well as those who have no agreements with them.

**5. Loss of interests in previous hobbies and entertainment as a result of, and**

**with the exception of, internet games** (IGD in DSM-5) – I**ncreasing priority given to gaming to the extent that gaming takes precedence over other life interests and daily activities** (GD in ICD-11)

This is the second criterion that is included in both the DSM-5 and the ICD-11, albeit in different formulations. The former includes four parts: (1a) “loss of interest”, (1b) “previous hobbies and entertainment”, (1c) “as a result of internet games”, and (1d) “with the exception of internet games”. The latter includes two parts: (2a) “increasing priority given to gaming”, (2b) “gaming takes precedence over other life interests and activities”. We start with the former again.

Due to the complex multi-part content of the IGD criterion, several different validity issues emerge here. Many of them relate the relationship between (1c) and (1d): by distinguishing between the two, the APA makes it clear that gaming should be both the reasons for the loss of interests (1c) as well as an ongoing activity that remains interesting (1d). Almost all instruments lack one or the other. For instance, PIE-9 states: “I have lost interest in previous hobbies and entertainment other than Internet games” (1d but no 1c).

Some items connect this criterion to other symptoms such social engagement, e.g. IGDS employs the following item: “have you been spending less time with friends, partner or family in order to play games?” In addition, an issue with items like this is that they do not inquire about loss of interest (1a), but time spent. The issue occurs in several instruments, e.g. CVAT 2.0: “Did you have to give up or strongly reduce important activities because of gaming? Examples: sports, work, or seeing friends/family.” Ultimately, we identified three instruments with relatively high content validity regarding this criterion. IGD Scale is missing (1d) but accurately includes everything else: “Have you lost interest in previous hobbies and leisure activities because of gaming?” The two remaining instruments also include (1d) and we have no critical comments on them: “Have you experienced loss of interests in previous hobbies and entertainment as a result of, and with the exceptions of, Internet games?” (C-IGDS) and “I have lost interest in other hobbies because of my gaming” (IGD-20).

Three of the four GD instruments fail to capture the element of increasing priority (2a) completely. GADIS-A asks: “I often do not pursue interests outside the digital world (e.g., meeting friends or partner in real life, attending sports clubs/societies, reading books, making music) because I prefer gaming.” While this question mixes up the “digital world” and “gaming”, it also does not include any of the temporality that is implied by the *increasing* priority. TIGTOC asks: “I have lost interest in other hobbies or recreational activities I enjoyed before because of Internet games” and GDHGS asks: “Do you prefer to play games rather than pursuing other hobbies and interests? Do you delay or avoid or ignore other daily activities to make time for gaming?” Again, (2a) is entirely missing from both. GDT, however, manages to capture (2a) well: “I have given increasing priority to gaming over other life interests and daily activities.” Even though the notion of precedence (2b) is merely hinted by the term “over”, we agreed this to be a high validity item.

**6. Continued excessive use of internet games despite knowledge of psychosocial problems** (IGD in DSM-5) – **Continuation or escalation of gaming despite the occurrence of negative consequences. The behaviour pattern is of sufficient severity to result in significant impairment in personal, family, social, educational, occupational or other important areas of functioning** (GD in ICD-11)

This is the third and final criterion, which is included by both the APA and the WHO in their respective diagnostic manuals. The DSM-5 description has two parts: (1a) “continued excessive use” that occurs (1b) “despite knowledge of psychosocial problems”. On the other hand, similar two components of (2a) “continuation or escalation of gaming” that happens (2b) “despite the occurrence of negative consequences” in the ICD-11 are supplemented by (2c) severity, negative consequences should be significant, and (2d) further explication of negative consequences in a form of personal, family, social, educational, occupational or other important areas of functioning. Visibly, the key difference between the two is that the former speaks of “psychosocial” problems in particular, which must also be “known”, where the latter “negative consequences” are more general and cover also other types of problems.

Whereas the content of some instruments has very little to do with the criterion to which they refer (e.g. IGD-20: “I often lose sleep because of long gaming sessions”), the foremost issue with the IGD description is its focus on “psychosocial” problems (1b), which are difficult to operationalize in practice. As a result of this pragmatic challenge, almost all instruments simply ignore this element and rather inquire about general problems, as in: “Due to my frequent gaming, I sometimes get in trouble at school or work” (CSAS), “Did you continue to play even though it created problems for you?” (IGD Scale), and “kept playing even though it caused problems” (IGD-BIC).

The only instrument that exactly captures (1a) and (1b) is C-IGDS: “Do you continue to use Internet games excessively despite knowledge of psychosocial problems?” Since the term “psychosocial” is likely difficult to interpret by the respondents, this may not be an optimal operationalization in practice – but we do not assess face validity here. IGUESS, in turn, divides psychosocial problems into two separate parts: “Despite social and psychological problems, I continue playing Internet games excessively.” However, social and psychological problems are not always connected directly and the mechanisms of their mutual dynamics are complex. As we discuss in the article, further clarity about the “psychosocial” element by the APA would help operationalization. Moreover, “playing games”, again, is not the same as “using games” (1a). Three more instruments (IGDC; IGDT-10; CVAT 2.0) give examples of encompassing negative consequences alone, without addressing “psychosocial” (1b).

Three of the GD instruments replicate the ICD-11 description (2a) and (2b) with little variation. GDT provides a statement: “I have continued gaming despite the occurrence of negative consequences” and TIGTOC is: “Despite social and psychological problems, I continue playing Internet games excessively.” GDHGS then asks: “Have you continued with gaming behaviour despite of the occurrence of negative consequences?” Severity (2c) and explication of consequences (2d), however, is present only in GDT’s fourth item using almost the same wording: “I have experienced significant problems in life (e.g., personal, family, social, education, occupational) due to the severity of my gaming behavior.” The fourth instrument, GADIS-A, in turn, is split into three items, which all try to capture impairment mostly in personal, family, and social areas of functioning: “I often continue gaming even though it causes me stress with others (e.g., my parents, siblings, friends, partner, teachers). Due to gaming, I neglect my appearance, my personal hygiene, and/or my health (e.g., sleep, nutrition, exercise). Due to gaming, I risk losing important relationships (friends, family, partner) or have lost them already.” Educational or occupational (2d) areas together with severity (2c) are omitted.

**7. Has deceived family members, therapists, or others regarding the amount of internet gaming** (IGD in DSM-5)

The seventh criterion in the DSM-5 concerns the deception of others. Three parts can be identified: (1a) “has deceived”, which concerns (1b) “family members, therapists, or others” and (1c) “regarding the amount of internet gaming”. Notably, almost all instruments use the term “lie” instead of “deceive”, which to a high degree can be considered synonymous but excludes all non-verbal forms of deception and requires intentionality (Adler 1997). For instance, IGDT-10 and IGD-BIC take such non-verbal deceptions into consideration by adding the phrase “keep others from knowing” to their item: “Have you tried to keep your family, friends or other important people from knowing how much you were gaming or have you lied to them regarding your gaming?” (former) or “kept others from knowing how much I play” (latter).

We found this criterion the most valid overall in terms of its operationalizations, and no less than six other instruments also accurately meet all three parts of the criterion. For instance, IGDS9-SF and C-IGDS use almost the exact same wordings, the former being “Have you deceived any of your family members, therapists or others because the amount of your gaming activity?” and the latter “Have you deceived family members, therapists, or others regarding the amount of Internet gaming?” PIE-9 uses the statement “I have deceived family members, therapists, or others regarding the amount of time I spend Internet gaming.” We do not have critical comments on these items.

**8. Use of Internet games to escape or relieve a negative mood (e.g., feelings of helplessness, guilt, anxiety)** (IGD in DSM-5)

The eighth criterion concerns mood moderation. This can be divided into (1a) the use of internet games, which is instrumental as either (1b) escape of negative mood or (1c) relief of negative mood. Three examples are given, i.e. feelings of helplessness, guilt, and anxiety. In this way, the criterion itself uses “moods” and “feelings” synonymously, and many instruments have chosen to apply only one of them. We do not pay attention to this issue since the DSM-5 is unclear. We again add that since the DSM-5 has chosen the word “use” of internet games, this could also include watching e.g., via live-stream gaming services. Few of the instruments have taken this into consideration, and the rest explicitly talk about “playing” internet games.

Notably, the word “mood” has often been replaced or represented by “problems” in the instruments. For instance, CVAT 2.0 asks: “Did you regularly play videogames to avoid thinking about problems (difficulties)” and CSAS asks “Playing games is the best way for me to forget about my problems” – both of which represent an utterly different content. Even more abstract representation of problems is used in the IGDS-23: “Did you play games more when other things in your life were not going well?”, which only indirectly suggests (1c). Seven instruments inquire about mood and feelings in line with the DSM-5 criteria (1a). Of these, two are missing the dimension of escape, as in IGDT-10: “Have you played to relieve a negative mood (for instance helplessness, guilt, or anxiety)?” and IGD Scale: “Did you play to reduce negative feelings (like helplessness, guilt, anxiety)?” The authors of IGUESS have chosen to replace “escape” with the word “avoid”: “I play Internet games to remove or avoid negative feelings.” The notion of avoidance, which is preventive, may result in higher prevalence rates.

Three instruments have high content validity. IGDS9-SF asks: “Do you play in order to temporarily escape or relieve a negative mood (e.g., helplessness, guilt, anxiety)?”, which corresponds well with the DSM-5 criterion, with the caveat of choosing the word “play” instead of “use”. The remaining two instruments, PIE-9 (“I use Internet games to escape or relieve a negative mood”) and C-IGDS (“Do you use Internet games as a way of escaping or relieving a negative mood (e.g., feeling of helplessness, guilt, anxiety)?”) both have high content validity.

**9. Has jeopardized or lost a significant relationship, job, or education or career opportunity because of participation in internet games** (IGD in DSM-5)

The final criterion of IGD includes several dimensions. First, the criterion offers two alternative events, i.e. (1a) jeopardizing and (1b) losing. The objects of jeopardization or loss are four, which must all be considered (1c) “significant” by the respondent: (1d) relationship, job, or education or career, furthermore, (1e) opportunities do count toward this list as well. None of the instruments capture this wide-ranging criterion to the full. CVAT 2.0 reduces the jeopardization or loss of the named significant entities into “problems”: “Did you play games even though you knew this was causing problems with your family, friends, at work, or at school?” IGDT-10, in turn, chooses to ask not about jeopardizing work but “work performance”: “Have you ever in the past 12 months jeopardized your school or work performance because of gaming?”

Seven instruments meet the basic content criteria of jeopardizing (1a) and loss (1b). The former is often replaced by “risking”, which we agree to be synonymous. For instance, CSAS states: “I have already lost or risked an important relationship or friendship because of gaming. Due to gaming, I have risked my opportunities at school or work.” For some reason, in this item only “risking” (but not loss) concerns school and work. In IGUESS, the career and educational opportunities (1d) are presented as opportunities in education and other domains: “I have risked or lost important opportunities in personal relationship, work, education or my career because of excessive Internet games playing.” In the IGDS-23 likewise: “Did you risk or lose an opportunity at school or work because of playing games?” Notably, there is a critical difference between a career opportunity (e.g. new job) and an opportunity that takes place in the career domain or work (e.g. having dinner with a colleague).

Five instruments have moderate to high content validity with this final criterion. IGD Scale does not use either of the DSM-5 terms (1a) or (1b), but is otherwise coherent: “Have you risked or ruined an important relationship, job, education or career opportunity because of gaming?” The only change made by IGDC, again, is replacing jeopardizing with risking: “Do you risk or lose significant relationships, or job, educational or career opportunities because of gaming?” The remaining four instruments use almost identical wordings, which include the exact content expressed by the ninth DSM-5 criterion. We have no critical comments on them.

**References**

Adler, J. E. (1997). Lying, deceiving, or falsely implicating. *The Journal of Philosophy*, 94(9), 435. doi:10.2307/2564617
